# Plant-Soil Feedbacks for the Restoration of Degraded Mine Lands: A Review

**DOI:** 10.3389/fmicb.2021.751794

**Published:** 2022-01-11

**Authors:** Shi-Chen Zhu, Hong-Xiang Zheng, Wen-Shen Liu, Chang Liu, Mei-Na Guo, Hermine Huot, Jean Louis Morel, Rong-Liang Qiu, Yuanqing Chao, Ye-Tao Tang

**Affiliations:** ^1^School of Environmental Science and Engineering, Sun Yat-sen University, Guangzhou, China; ^2^Guangdong Provincial Key Laboratory of Environmental Pollution Control and Remediation Technology, Guangzhou, China; ^3^Guangdong Provincial Engineering Research Center for Heavy Metal Contaminated Soil Remediation, Sun Yat-sen University, Guangzhou, China; ^4^Laboratoire Sols et Environnement, INRAE-Universiteì de Lorraine, Vandoeuvre-leÌs-Nancy, France; ^5^CNRS, LIEC, Université de Lorraine, Nancy, France; ^6^Guangdong Provincial Key Laboratory of Agricultural and Rural Pollution Abatement and Environmental Safety, College of Natural Resources and Environment, South China Agricultural University, Guangzhou, China; ^7^Guangdong Laboratory for Lingnan Modern Agriculture, Guangzhou, China

**Keywords:** plant-soil interactions, degraded mine lands, ecological restoration, plant functional traits, ecosystem functions, soil community

## Abstract

Much effort has been made to remediate the degraded mine lands that bring severe impacts to the natural environments. However, it remains unclear what drives the recovery of biodiversity and ecosystem functions, making the restoration of these fragile ecosystems a big challenge. The interactions among plant species, soil communities, and abiotic conditions, i.e., plant-soil feedbacks (PSFs), significantly influence vegetation development, plant community structure, and ultimately regulate the recovery of ecosystem multi-functionality. Here, we present a conceptual framework concerning PSFs patterns and potential mechanisms in degraded mine lands. Different from healthy ecosystems, mine lands are generally featured with harsh physical and chemical properties, which may have different PSFs and should be considered during the restoration. Usually, pioneer plants colonized in the mine lands can adapt to the stressful environment by forming tolerant functional traits and gathering specific soil microbial communities. Understanding the mechanisms of PSFs would enhance our ability to predict and alter both the composition of above- and below-ground communities, and improve the recovery of ecosystem functions in degraded mine lands. Finally, we put forward some challenges of the current PSFs study and discuss avenues for further research in the ecological restoration of degraded mine lands.

## Introduction

In the terrestrial ecosystem, plants alter their surrounding biotic and abiotic soil conditions through root- and litter-induced effects, which in turn influence the growth, productivity, and generation of the coexistent and subsequent plants. This is called plant-soil feedbacks (PSFs) (Wardle et al., 2004; van der Putten et al., 2013). Increasing attention has been paid to PSFs due to their contribution to plant community dynamics and ecosystem functioning regulation. Until now, most of the studies on plant-soil interactions have focused on uncontaminated natural systems, like tropical forests and temperate grasslands, or agricultural systems (Mariotte et al., 2018). In those ecosystems, most PSFs researches aim to understand the process of plant succession dynamics (Kardol et al., 2006, 2013; van de Voorde et al., 2011), plant expansion and invasion (van Grunsven et al., 2010), plant community composition maintaining (Mangan et al., 2010; Dassen et al., 2017; Teste et al., 2017), and biotic responses toward human-induced global land use and climate changes (Geisen et al., 2017; Fry et al., 2018a; Pugnaire et al., 2019). Even though the degraded mine lands pose obvious threats to the environment, relatively few studies have focused on the PSFs at these sites (Krumins et al., 2015; Jia et al., 2020). According to the facilitating or inhibiting effects toward the adaptive performance and monopolization of conspecifics, PSFs are considered positive, negative, or neutral (van der Putten et al., 2013). Generally, PSFs is considered positive when it improves the growth of contemporaneous or later plants. The PSFs direction may have significant differences during primary and secondary succession. For example, at the beginning of primary succession, positive feedbacks by nursing plants are critical for ameliorating the harsh conditions and motivating the natural colonization of multiple plant species (Reynolds et al., 2003). While in the earlier stages of a secondary succession that starts with a certain soil legacy, negative PSFs caused by rhizosphere pathogen can stimulate species turnover (Kardol et al., 2013; Cortois et al., 2016). However, compared to studies in secondary succession, the knowledge of PSFs patterns and driving factors during the primary succession in mine lands is still in its infancy.

Degraded mine lands are generally provided with a sparse density of vegetation, soil compaction and acidification, heavy metal toxicity, and nutrient deficiency (Boyer and Wratten, 2010; Hu et al., 2012), which may alter the dynamics and outcomes of plant-soil interactions. In order to colonize in mine lands, tolerant plants should be equipped with some species-specific functional traits to adapt to extreme conditions, thus inducing more uncertainties to the performance of PSFs. Soil communities (e.g., bacteria, fungi, protists, and invertebrates) play key roles in influencing the soil environment, buffering plant individuals and harsh soil abiotic conditions, and altering the composition of the above-ground community. To recover biodiversity and ecosystem multifunctionality, we need to explore and follow the laws of nature. A better understanding of the patterns and driving factors of PSFs during the ecological restoration processes would help us to accelerate the recovery of vegetation and soil properties and finally achieve ecosystem multifunctionality.

Here, we address plant-soil feedbacks based on soil properties, plant functional traits, and soil communities in contaminated mine tailings and discuss their consequences for the ecosystem functioning and potential remediation practices. We first describe the current situation and obstacles for ecological restoration of mine lands, then focus on how plant and soil communities interact under harsh conditions. We also review the litter- and rhizosphere-induced PSFs based on multiple plant functional traits and the interactions with associated microorganisms for the tolerant plants established in mine areas. Moreover, we discuss how the understanding of PSFs plays a pivotal role in accelerating the recovery of ecosystem functions including pollutant control, primary productivity, decomposition, nutrient availability, and cycling. In the end, we summarize the challenges and prospects of PSFs study in abandoned mining areas, emphasizing more efforts should be made to fulfill the imperative knowledge and practical gaps.

## Current Situations of Mine Lands Restoration

Mining activities not only clear above-ground vegetation and damage soil communities but also leave behind large areas of waste, i.e., tailings (Bradshaw, 1997; Hu et al., 2012). These lands are usually featured with compaction, erosion, and desertification of soil, accumulation and pollution of toxic metals, deficiency of nutrient and organic matter, and loss of biodiversity (Boyer and Wratten, 2010; Hu et al., 2012; Wang et al., 2018). Therefore, the development of ecosystems in mine lands can be categorized as a primary succession that commonly needs 50 to 100 years to gain a fundamental vegetation establishment *via* natural processes (Bradshaw, 1997; Li and Liber, 2018). Consequently, different kinds of human interventions (Gastauer et al., 2018) have been developed to accelerate ecological recovery. However, there are still many problems that need to be further considered.

When conducting the restoration of degraded mine lands, it is imperative to improve the physical and chemical environment, eliminate the threats to life health, recover the assemblage of functional species groups, and ultimately achieve the structurally and functionally self-sustaining ecosystems (Society For Ecological Restoration International Science [SER], and Policy Working Group, 2004; Lei et al., 2016). One of the most effective ways to attenuate the soil quality and address ecological restoration is revegetation in such barren lands (Mukhopadhyay et al., 2017; Li and Liber, 2018). However, because of the low level of soil available nutrients and high level of metal toxicity, few plant species can survive in these areas (Li and Liber, 2018; Wang et al., 2018). Additionally, the selection of promising plants is difficult when considering biological invasion risks (Gaertner et al., 2012; Nussbaumer et al., 2016). Some passive or improper managements probably import invasive species at the beginning of restoration (Bauman et al., 2015), while they may hinder the colonization of native species (Davis et al., 2005), resulting in low biodiversity and an unstable ecosystem (Schwenke et al., 2000; Hamman and Hawkes, 2013; Bauman et al., 2015). Furthermore, numerous restoration projects failed to meet the objective of environmental sustainability (Lamb et al., 2015), as the trajectory of succession altered and the diversity of reconstructed plants community declined with time (Schneemann and McElhinny, 2012). For example, the improper combination of species and revegetation management like continual sowing enhanced individual competition and limited the survival of some species (Neldner and Ngugi, 2017).

During the ecological restoration of degraded mine lands, the assistance of the below-ground community cannot be ignored (Grandlic et al., 2009; Rajkumar et al., 2012; Hao et al., 2014; Ullah et al., 2015; Wang, 2017). As an important component of the ecosystem, soil organisms affect terrestrial ecosystem functions by regulating soil acidity, mediating soil carbon dynamic, stabilizing toxic pollutants, and promoting element cycling (Chen et al., 2016; Fierer, 2017; Sun et al., 2017, 2018b). In a mine land co-contaminated with arsenic (As) and antimony (Sb), the potential tolerant bacteria *Bradyrhizobium*, *Sphingomonas*, *Nocardioides*, *Burkholderia*, and *Streptomyces* induced As and Sb biogeochemical cycling, as well as contributed to the cycling of C (C fixation), N (nitrate/nitrite, N fixation), and S (sulfate reduction) (Li et al., 2021). The rhizospheric microbes serve as an important assistor for plants to survive in harsh environments (Grandlic et al., 2009; Mishra et al., 2017; Sun et al., 2018b). However, the survival and composition of soil microbiome are largely dependent on soil environment factors like soil pH (Lauber et al., 2009), available nitrogen (Cederlund et al., 2014), soil organic carbon (Sul et al., 2013), redox status (Pett-Ridge and Firestone, 2005), and especially heavy metal content and availability (Singh et al., 2019). Technical problems of improper bacteria carriers and poor production of bacteria biomass can lead to insufficient buildup and rapid decline of the plant growth promoting bacteria (PGPB) population during practical inoculation (Bashan et al., 2014). Additionally, it is more efficient to formulate and apply multi-strain inoculants compared to single-strain ones, but the premise is that we have a thorough understanding of the adaptability of microorganisms to the stress environment, the compatibility among microbial populations, and the symbiosis and interaction with plants (Bashan et al., 2014).

Plenty of studies have utilized plant-microbe association in mine lands bioremediation, but few of them have figured out how PSFs influence ecological succession, leading to incomplete and unsustainable remediation. As the intensive interactions and complexity of cooperation among plants and soil biotic and abiotic components (Krumins et al., 2015), it is usually inefficient to execute restoration schemes for degraded mine lands without addressing the underlying processes and mechanisms of above-below ground linkage (Li and Liber, 2018). Only the mechanisms of plant-soil interactions in degraded mine lands are revealed, can we make significant progress on precisely ameliorating stressed conditions in mine areas and facilitating the restoration of the plant community and even the whole ecosystem functioning.

## Plant-Soil Feedbacks in Degraded Mine Lands

**FIGURE 1:**
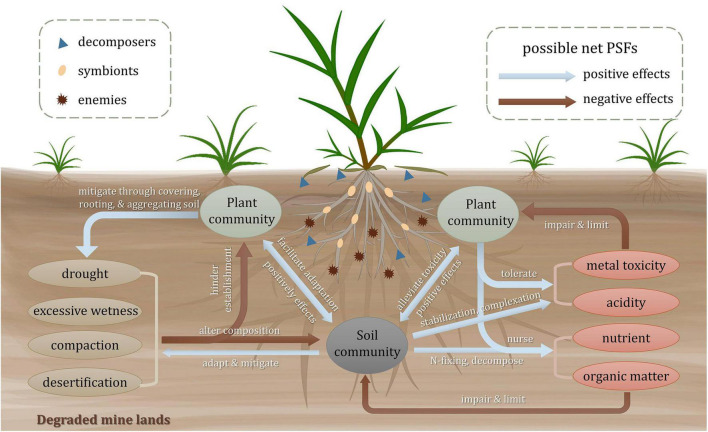
Plant-soil interactions in degraded mine lands. Arrows in the plant-soil systems present the possible net plant-soil feedbacks among soil abiotic properties, plant and soil community, with red arrows for negative feedbacks and blue arrows for positive feedbacks. For ellipses in different colors, green ones represent plant communities, gray ones represent soil community, yellow ones represent physical properties, and red ones represent chemical and biogeochemical properties, respectively. Different graphics in the plant rhizosphere represent three groups of soil biota including enemies, symbionts, and decomposers depending on different shapes.

Plant-soil feedbacks can be affected by soil physical, chemical, and biogeochemical properties. As shown in Figure 1, the severe soil properties of the degraded mine lands impede the establishment of plant community and alter the composition of the soil biological community. Once the pioneers colonize, the plants and microbes, in turn, ameliorate the soil conditions. Meanwhile, a mutual influence also exists between plants and soil communities (Krumins et al., 2015). Generally, plant-associated soil biota that shapes PSFs can be divided into three categories: enemies like microbial pathogens, symbionts like mycorrhizal fungi and nitrogen-fixing rhizobia, and decomposers like saprophytes (van der Putten et al., 2016). They are the main biotic determinants of the direction (positive, neutral, or negative) and the strength (mild or strong) of PSFs (Wardle, 2013; van der Putten et al., 2016). The pioneer plants equipped with special functional traits are experiencing and modifying the environment with those associated soil biota. The possible responses of the PSFs toward these extreme factors are reviewed as follows.

### Responses of Plant-Soil Feedbacks Toward Extreme Conditions

#### Physical Properties

Due to the scattered or bare vegetation, the soils of the degraded mine lands are always compacted and suffer from either drought or excessive wetness (Bradshaw, 1983). Both too dry and too wet conditions impair plant growth and soil biota survival (Bradshaw, 1997). It has been reported that changes in soil community composition induced by drought can cause negative PSFs to plant species and finally alter plant-plant interactions (Kaisermann et al., 2017). The soil compaction usually produces hardened layers and anaerobic conditions, which is not conducive for plant rooting and brings negative feedbacks to the plant and soil community (Boyer and Wratten, 2010). Besides, the desertification of tailing soil probably causes soil erosion and hinders plant establishment (Zhou et al., 2015). However, some pioneer soil organisms like soil fauna and microorganisms may induce positive PSFs. Among the soil fauna, earthworms are important candidates for attenuating compaction and water retention conditions. They can increase soil macroporosity and soil aggregation by burrowing activity (Boyer and Wratten, 2010). The dry-adapted microorganisms tend to mitigate the drought effects and facilitate seed germination, seedling development, and biomass production (O’Brien et al., 2018). The soil physical stresses tend to be gradually mitigated by plant covering, rooting, and aggregating functions (Li et al., 2013).

#### Chemical and Biogeochemical Properties

Metal toxicity is one of the most critical factors limiting above- and below-ground lives (Kang et al., 2020). Negative correlations have been found between soil microbial activity and soil extractable heavy metal concentrations (Wang et al., 2007). It is supposed that the capability of plant tolerance to toxic metals and the acceptance of positive feedbacks from soil mutualisms determine the existence and composition of plant communities (Krumins et al., 2015). Tolerant plants may attract and foster oligotrophic and metal-tolerant communities in the rhizosphere through a variety of organic and inorganic exudates (Borymski et al., 2018). Rhizosphere microorganisms would alleviate the detrimental effects of metal toxicity for plants through some biological processes, including complexation by organic acids and siderophores (Schalk et al., 2011), stabilization by secreting extracellular polymeric substances (Ahonen-Jonnarth et al., 2000; Joshi and Juwarkar, 2009), and increasing plant antioxidative defense by producing 1-aminocyclopropane-1-carboxylic acid (ACC) deaminase (Gao et al., 2010). Low pH stress in mine lands can cause negative feedbacks to the biotic community directly and indirectly. For one thing, the research on a massive copper mine tailings showed that the increase of acidity arouses the reduction of overall microbiome diversity and relative abundance of dominant assemblages (Liu et al., 2014; Shanmugam and Kingery, 2018). The abundance of soil protozoan taxa is also proved to be regulated by soil pH (Xu et al., 2022). For another, the availability of metals in acid soils is often higher than those in neutral or calcareous soil, which enhances the toxic effect of heavy metals on plant and soil communities. The lack of nutrients and organic matter limits the development of plant communities (Li and Liber, 2018). Some mutualistic symbioses like arbuscular mycorrhizal fungi (AMF) can provide significant assistance for plants to deal with nutrient deficiency *via* AM nutrient uptake pathway (Smith et al., 2010). The nitrogen supplied by diazotrophic communities facilitates the establishment of microbial and the growth of plants in tailings (Sun et al., 2020). The presence of nursing plants like legumes can increase the input of nitrogen through the N-fixing capacity of rhizobia in the rhizosphere, which improves below-ground nitrogen cycling and brings positive feedbacks for later successional plants (del Moral and Rozzell, 2005; Ren et al., 2008). Pioneer plants tend to modify soil fertility conditions beneath their canopy by litter input, which may form vegetated patches and nutrient islands (Navarro-Cano et al., 2018). Additionally, the positive effects between biodiversity and nutrient availability play an important role in ecological succession in degraded mine lands (Wang et al., 2018).

### Features of Plant-Soil Feedbacks in Mine Lands

Compared to secondary successional (Kardol et al., 2013) and other multi-species contexts like tropical forest (Mangan et al., 2010) and old-field (van de Voorde et al., 2011), positive PSFs may outweigh the negative ones in the early stage of primary successional ecosystems for further development of plant community and restoration of ecosystem functioning (Reynolds et al., 2003; Ehrenfeld et al., 2005; Krumins et al., 2015). According to the stress gradient hypothesis, the variance between facilitation and competition of plant interactions will change along the environmental stress gradient, with positive feedbacks dominating under high abiotic stress (Travis et al., 2006; He et al., 2013; Song et al., 2019). It is supposed that in a harsh environment, the pioneer plants tend to buffer and modify the biotic and abiotic stresses for adjacent plants (Bertness and Callaway, 1994). Thus, it is legitimate to suppose that positive feedbacks may exist in early successional mine lands and, with the extreme conditions being gradually attenuated, finally give way to negative feedbacks along the primary successional trajectory (Reynolds et al., 2003; Krumins et al., 2015). However, this supposition is waiting to be confirmed due to the scarcity of relative long-term empirical evidence in these degraded ecosystems.

The harsh physicochemical properties in mine lands determine whether and which plants can survive and make a difference (Ahirwal et al., 2017). So the soil properties act as both important influencing and affected factors in PSFs. From the angle of influencing factors, it is critical to evaluate the dominant soil properties in the PSFs process. From the angle of affected factors, plants produce biotic and abiotic legacy in the soil, which will influence plants for a long period. And the succession of the mine land ecosystem needs to be triggered by the colonization of tolerant lives and their following PSFs. The sum of positive and negative feedbacks i.e., net PSFs, can indicate the direction of succession trajectory, and the strengths of the PSFs can determine the velocity of natural succession. However, the truth and mechanisms about the critical biotic factors altering PSFs direction and strengths in mine lands are still puzzling us out there and waiting to be figured out.

## Ecological Strategies of Tolerant Plants

### Functional Traits of Tolerant Plants

**FIGURE 2:**
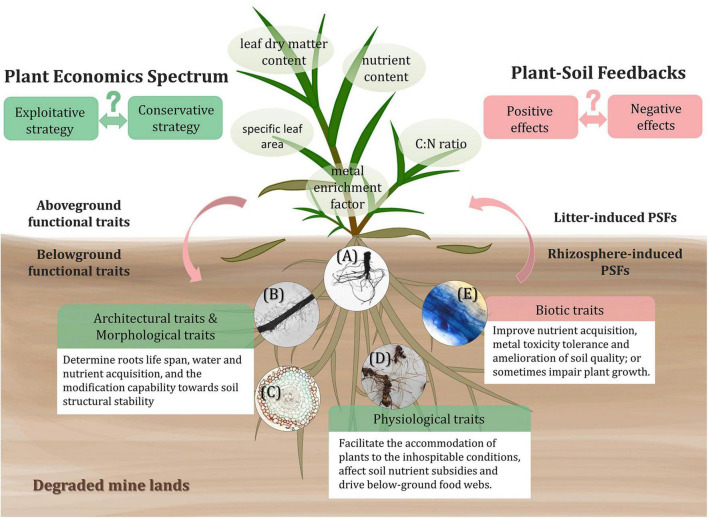
Above- and below-ground functional traits of tolerant plants inducing PSFs in degraded mine lands. The trade-offs of functional traits economics spectrum and the patterns of PSFs of different plants toward severe conditions can alter the ecosystem functioning and are yet to be explored. As for belowground traits, the architectural traits include root depth, root branching; morphological traits contain root diameter, specific root length; physiological traits consist of nutrient content, root respiration, root exudation; and biotic traits refer to the interactions between roots and AMF, rhizobia, PGPB, and pathogens. The shown circular images: **(A)** root system scanning image of *Phytolacca americana*, representing root architectural traits; **(B)** the root segment of *Digitaria sanguinalis* and **(C)** the cross-section of *Dicranopteris linearis* root, both representing morphological traits; **(D)** the rhizome and fine roots of *Miscanthus sinensis*, representing roots nutrient content, i.e., physiological traits; and **(E)** the stained root segment of *Miscanthus sinensis* colonized with AMF, representing biotic traits.

Plant functional traits, defined as the measurable features that determine plants performance or fitness, include morphological, behavioral, and biochemical attributes (Reich et al., 2003; Cadotte et al., 2011; Figure 2). Species that establish in mine lands tend to possess some self-suited resistant traits and functions to conquer the environmental stress (Nirola et al., 2016b; Quintela-Sabaris et al., 2020). Plant functional traits can modify the soil environment and influence the composition of the soil microbial community (Fierer, 2017), thus playing an important role in understanding the PSFs of different plant species when confronting environmental stress and interferences (Colin et al., 2019).

Above-ground functional traits like specific leaf area, leaf dry matter content, nutrient content, and C:N ratio are frequently characterized to reveal the above-ground response of plants to environmental conditions (Schroeder-Georgi et al., 2016; Veen et al., 2019; Quintela-Sabaris et al., 2020). Depending on different leaf traits, the input of plant foliar litter can be beneficial or detrimental to the soil environment and even to the later plant generations (van der Putten, 1997; Veen et al., 2019), which is also regarded as litter-induced PSFs (Kardol et al., 2015). For example, in relatively infertile conditions, the carbon and tissue nutrient concentrations of litter are the key drivers of soil nutrients and organic matter pool (Wardle et al., 2004). The fast-growing or exploitative plants tend to produce high-quality litters relatively rich in nutrients, stimulating a high decomposing rate (Gartner and Cardon, 2004; Parker et al., 2018), from which the later plant species can receive more positive feedbacks (Wardle et al., 2004). However, research on reclamation in an ultramafic degraded area presented opposite results, that is, perennial plant species with a conservative growth strategy are more suitable for the reclamation since they can better adapt to the extremely low available nutrient condition (Quintela-Sabaris et al., 2020). Thus, the economics spectrum patterns of tolerant plants for phytoremediation on different degraded soil still require particular research attention. In heavy metal contaminated mine lands, the enrichment factor of metallic elements in the aerial part is also an important trait (Nirola et al., 2016a). The leaves with high metal concentration may deposit in the soil beneath their canopies and result in the reallocation of available metal toxicity on the upper soil layer (Colin et al., 2019; Reynolds et al., 2020), bringing negative effects to other relatively heavy metal sensitive plants. The accumulation of toxic elements by hyperaccumulator plants also tends to cause harmful effects on decomposing organism community and retard soil carbon and nutrients cycling (Hooda, 2010). It is necessary to consider the balance between the benefit of hyper-accumulating effects and the accompanying ecological influence when applying phytoremediation measures.

The below-ground functional traits we discuss here mainly refer to plenty of root-related traits, including architectural, morphological, and physiological traits. These traits significantly mediate the soil organisms and alter interactions between plant and soil, so-called rhizosphere-induced PSFs (Bardgett et al., 2014; Kardol et al., 2015). For instance, the architectural (e.g., root depth and root branching) and morphological (e.g., root diameter and specific root length) traits determine the life span of roots, the acquisition of water and nutrient (Dunbabin et al., 2004; Colin et al., 2019), and the modification capability toward soil structural stability (Gyssels et al., 2005; Bardgett et al., 2014). A research conducted in abandoned metal mine deposits shows that plant root architecture and morphological structure are among the key factors that condition the diversity and structure of soil bacteria during primary succession (Colin et al., 2019). For adaptation, the trade-offs between source acquisition and environmental stress tolerance, i.e., root economics spectrum, commonly exist among plants (Chave et al., 2009; Lin et al., 2019). Particularly, exploitive species with high specific root length and low tissue density tend to have a higher rate of water and nutrient uptake, however, they are easier to experience negative PSFs due to lower root resistance strength and short life span and *vice versa* (Valenzuela-Estrada et al., 2008; Freschet and Roumet, 2017). The physiological traits (e.g., nutrient content, root respiration, and root exudation) also help plants to accommodate the inhospitable condition, affect soil elemental stoichiometry and drive below-ground food webs (Hines et al., 2006; Zechmeister-Boltenstern et al., 2015; Zhang et al., 2016). For example, the carbon input is capable of stimulating microbial activity and boosting the uptake and immobilization of nutrients by microbes (Zhang et al., 2016). Fine root debris with higher nitrogen content decomposes faster and provides more nutrients for plant and microbial growth (Kardol et al., 2015).

In general, exploitative plant traits (high specific leaf area and leaf N) are more related to a microbial community dominated by bacteria compared with that by fungi (de Vries et al., 2012), leading to more energetic nutrient circulation. Consequently, an increasing number of researchers adopt trait-based approaches to address plant identity effects on ecosystem developing trajectory based on PSFs (Kardol et al., 2015; Kraft et al., 2015; Cortois et al., 2016; Schroeder-Georgi et al., 2016; Colin et al., 2019). In trait-based ecology, the trade-offs of the plant economics spectrum between resource acquisition and conservation are one of the most important issues (Shen et al., 2019). Within the context of degraded mine lands, the plant functional trait approaches are initially used for the selection of plants for restoration (Ilunga wa Ilunga et al., 2015; Quintela-Sabaris et al., 2020). Take a study in Katangan mine land for instance, with the purpose of revegetation on bare soils in metal-rich lands, annual plants that grow in the wet season and have a deep root system can be chosen as the candidates (Ilunga wa Ilunga et al., 2015). While when considering the vegetation functional biodiversity fulfilling and heavy metal phytostabilization enhancing, it is better to select cespitous grass species with dense rooting mat to avoid long-period bare soil during the dry season (Shutcha et al., 2010). It is of great importance for the restoration of similar situations in mine lands since the appropriate traits category is a more universal criterion than the species pool (Ilunga wa Ilunga et al., 2015).

### Roles of Soil Microbial Community in Assisting Plants Performance

The interaction and symbiosis between plants and rhizospheric microbes sometimes are also considered as root biotic traits of plants (Figure 2), which act as critical determinants altering PSFs (Bardgett et al., 2014). The associating below-ground microbial community involves AMF, rhizobia, PGPB, and pathogens (Rajkumar et al., 2012; van der Putten et al., 2016). Most plants growing in mine lands are primarily colonized by AMF in the roots, which allow them to receive positive feedbacks (Quoreshi, 2008) *via* improvement of nutrient acquisition (Smith et al., 2003), enhancement of metal toxicity tolerance (Miransari, 2010), and amelioration of soil quality and structure (Madiba, 2014; Wang, 2017). The large surface area of extraradical hyphae, high affinity and transporting speed toward nutrient elements enable the AMF to assist nutrition acquisition by host plants (Wang, 2017). The AMF could also relieve the heavy metal toxicities to plants through chelating or immobilizing toxic metals by extraradical mycelium (González-Guerrero et al., 2009). Some prokaryotic microorganisms containing nitrogen fixation genes are metal resistant and important for the improvement of N availability in mine lands (Sun et al., 2018a). Rhizobia are also a kind of beneficial microbes that can facilitate the growth of plants by not only fixing nitrogen but also mitigating metal toxicity by increasing metal isolation in root nodules, providing positive PSFs in the rhizosphere of legumes (Hao et al., 2014). Additionally, positive feedbacks are also mediated by rhizosphere PGPB, which excrete multiple metabolites like indole-3-acetic acid (IAA), siderophores, organic acids, and extracellular enzymes, etc (Rajkumar et al., 2012; Mishra et al., 2017). However, except for these beneficial microbes, harmful enemies like pathogens are inevitably dispersed in the plant-soil system, inducing negative feedbacks. For example, during early successional stages, plants with lower specific root lengths are more susceptible to fungal pathogens, and leading to a higher rate of negative inhibition for plant growth (Bergmann et al., 2016).

Together, though relevant researches are increasing, what we know about how plant functional traits and soil microbes facilitate plant growth in degraded mine lands is still far from enough. The integration of shoot and root functional traits and the associated soil communities can help understand the ecological strategy of tolerant plants in driving PSFs in mine lands and select ideal plant functional groups to trigger and accelerate the natural succession process. Digging into the functioning of soil communities on assisting plants performance in mine lands may disentangle the complexity of PSFs and improve the application of bioremediation in the future.

## Recovery of Ecosystem Functions

To evaluate the consequences of the ecological restoration in degraded lands (Fry et al., 2018b; Jiao et al., 2019), increasing attention has been focused on ecosystem multifunctionality (Sanderson et al., 2004). These ecosystem functions include pollutant control, primary productivity, decomposition, nutrient availability, and cycling, etc (Loreau et al., 2001; Society For Ecological Restoration International Science [SER], and Policy Working Group, 2004; Delgado-Baquerizo et al., 2020). The successfully restored systems can maintain sustainability with minimal need for extra assistance (Society For Ecological Restoration International Science [SER], and Policy Working Group, 2004; Walker and Moral, 2009). Meta-analyses showed that biodiversity is positively correlated with ecosystem functions in most cases (Cardinale et al., 2006; Gross et al., 2014). Thus, achieving more predictable consequences of biodiversity and ecosystem functions recovery turns out to be one of the most important goals in restoration ecology (Matthews and Spyreas, 2010). Up to date, the most practically suitable approaches for mining ecosystem restoration are still under discussion and research.

Functional trait-based approaches play important roles in interpreting the variability of biodiversity and multifunctionality recovery (Zirbel et al., 2017). With a brighter understanding of PSFs outcomes in different types of degraded mine lands, we can apply the mechanisms on selecting the suitable tolerant plant species for revegetation and biodiversity recovery. At the outset of primary succession, nurse plants play an important role in facilitating the colonization and reproduction of later plants (Foronda et al., 2020). In one case, during the restoration of ecosystem functions under extremely stressful mine tailings, some C4 plants with above-ground leaves of lower C:N ratio, acting as potential nurse plants, can improve soil TOC, N, and P content effectively (Navarro-Cano et al., 2018). In another case, the legume community of *Ononis tridentate* L. can form patchy fertility islands beneath its canopy on bare gypsum substrates *via* the symbiosis with below-ground N-fixing rhizobia (Navarro-Cano et al., 2015). Hence, the trait-based selection of nurse plants for remediating degraded mine lands is of great efficiency for long-term recovery. The applications of nurse plants to accelerate post-mining ecological restoration are considered as one of the environmentally friendly and sustainable nature-based solutions.

Soil communities also serve as an important factor driving plant diversity and ecosystem multifunctionality (Wagg et al., 2014; Delgado-Baquerizo et al., 2016). Empirical evidence shows that multiple metabolites like siderophores, IAA, and organic acids excreted by rhizosphere microbes can enhance plant functions of metal detoxification, nutrient uptake, and other biotic and abiotic stress mitigation (Rajkumar et al., 2012). The appropriate application of PGPBs and AMF for enhancing phytoremediation based on PSFs outcomes should be helpful for plant survival and biodiversity increase. Additionally, soil decomposers can ease plant competition by enlarging their habitats chemically (accelerate litter decomposition and improve nutrient uptake) and spatially (deepen plant roots) (Eisenhauer et al., 2012). Regulating the diversity of below-ground decomposers can effectively alter the effect of plant biodiversity on primary production (Eisenhauer et al., 2012). To sum up, by revealing the plant-soil system “black box,” it is practicable to use the mechanisms of PSFs to manipulate the trajectory and rate of plant community succession and ecosystem functions recovery. However, cases of applying PSFs mechanisms in natural degraded system recovery are far less than those in other relatively healthy systems (van de Voorde et al., 2011).

## Conclusion and Perspectives

In degraded mine land systems, the terminal objective is to restore a stable and sustainable ecosystem with abundant species and multifunctionality. Figuring out the possible patterns and underlying mechanisms of PSFs in mine lands is significant for understanding and predicting the process of succession under these barren toxic conditions. It is also critical for applying the PSFs mechanisms and functional trait-based approaches in nature-based solutions to accelerate the remediation. Although ecological researchers gradually focus on plant-soil systems of various environments, a large research gap remains in the restoration of these harsh environments. Here we put forward the perspectives and challenges of PSFs in degraded mine areas that require further research.

Firstly, studies about PSFs in degraded mine lands are necessary. As primary succession sites, degraded mine lands possess few biotic lives and little abiotic material legacy. It is an ideal scene to study the impact of PSFs on the initial formation and interactions of above- and below-ground communities. However, the establishment and regeneration of plant communities and the gathering of multiple species in mine lands are difficult and always take a long time, making the field study difficult. Simultaneously, manipulative experiments in the laboratory are also necessary for precisely understanding the patterns and the influence factors of PSFs. But the problem is that it is hard to fully simulate the condition in the wild. For example, intermittent drought is common in most mine lands, but the lab-grown plant may wither if they are not watered continuously and then we will not even have the biomass for PSFs calculation. Thus, a big challenge for researchers is to think over and make the choice of proper cultivating conditions between better for plant growth and simulating the true field environment.

Secondly, it is important to find out the key environmental factors and mechanisms that determine plant community succession in mine lands. Lots of abiotic stresses impede plant growth, but the critical abiotic factors that determine PSFs remain unknown. Moreover, the response of PSFs is usually context- and time-dependent. Identifying the most important factors that alter or hinder the primary succession can assist restoration efforts in degraded mine lands.

Thirdly, the economic spectrum of tolerant plant functional traits is waiting to be figured out. For one thing, knowing the functional traits that plants are equipped to overcome harsh environments can help to understand the plant economics spectrum along with environmental stresses and provide insights into a trait-based selection of candidate plant species for the restoration of mining sites. For another, plant functional traits are also useful to indicate the relationships between plants and soil community and predict plant effects on succession processing and ecosystem functioning.

Fourthly, it is necessary to combine the structure and functioning of the soil community with the performance of tolerant plants in mine lands to enhance phytoremediation. More emphasis is required on the development of trait-assisted and belowground-derived recovery approaches based on theoretical mechanisms of PSFs. We should surmount the obstacles along the way to revealing PSFs mechanisms and their application on the restoration. This may lead to an important complement to the theory of restoration ecology.

## Author Contributions

S-CZ and Y-TT conceived the ideas. H-XZ and W-SL provided the figures. S-CZ wrote the first draft of the manuscript. W-SL, Y-TT, YC, and HH reversed the manuscript. All authors contributed to manuscript revision, read, and approved the submitted version.

## Conflict of Interest

The authors declare that the research was conducted in the absence of any commercial or financial relationships that could be construed as a potential conflict of interest.

## Publisher’s Note

All claims expressed in this article are solely those of the authors and do not necessarily represent those of their affiliated organizations, or those of the publisher, the editors and the reviewers. Any product that may be evaluated in this article, or claim that may be made by its manufacturer, is not guaranteed or endorsed by the publisher.
